# Risk identification and improvement strategies for BMI and physical fitness and health grade cross-classification: a cross-sectional study based on Chinese college students

**DOI:** 10.3389/fpubh.2025.1660686

**Published:** 2025-10-30

**Authors:** Hao Guo

**Affiliations:** Zhumadian Preschool Education College, Economic Development Zone, Zhumadian, Henan, China

**Keywords:** physical fitness and health, body mass index, cross-classification, risk identification, intervention strategies, university students

## Abstract

**Introduction:**

The decline in physical fitness among university students has become a global concern. Traditional single-metric evaluation methods, such as body mass index (BMI) alone, cannot comprehensively capture students’ health status. This study aimed to establish a BMI–Physical Fitness and Health (PFAH) cross-classification framework to identify distinct risk groups and their characteristics, providing evidence for targeted intervention strategies.

**Methods:**

A cross-sectional study was conducted among 3,026 Chinese college students (1,435 males, 1,591 females; aged 18–22 years) assessed using the National Student Physical Fitness and Health Standard. Participants were cross-classified into eight groups according to BMI categories (normal, overweight, obese) and PFAH levels (good, pass, fail). Heat maps, radar charts, and receiver operating characteristic (ROC) curve analyses were used to visualize group features and identify key predictive indicators.

**Results:**

The most prevalent group was normal-pass (52.1%), followed by normal-good (22.2%) and overweight-pass (11.6%). High-risk groups—obesity-pass, normal-fail, overweight-fail, and obesity-fail—accounted for 3.0%, 4.7%, 2.5%, and 3.0% of the sample, respectively. Each high-risk group exhibited distinct fitness deficiencies: the obesity-pass group had significantly elevated BMI (z = 2.45); the normal-fail group showed poor speed (50 m: z = 0.69); the overweight-fail group displayed reduced flexibility (sit-and-reach: z = –0.47) and muscular endurance (sit-ups: z = –0.45); and the obesity-fail group performed worst in cardiopulmonary endurance (1,000 m: z = 1.60; 800 m: z = 2.30) and muscular strength (pull-ups: z = –0.86). BMI, endurance (1,000 m/800 m), and speed (50 m) were the strongest predictors for identifying high-risk males (AUC = 0.902, 0.801, 0.792) and females (AUC = 0.895, 0.874, 0.731).

**Discussion:**

The BMI–PFAH cross-classification framework effectively distinguishes diverse risk profiles among university students, revealing hidden risk populations (normal BMI but failing fitness) that traditional BMI-based assessments might overlook. Based on these findings, targeted interventions should include weight management while maintaining fitness for the obesity-pass group, anaerobic and speed training for the normal-fail group, and comprehensive improvements in cardiopulmonary function, muscular strength, and flexibility for overweight/obesity-fail groups. This framework provides a practical basis for developing evidence-based health education and personalized intervention strategies in higher education settings.

## Introduction

The global prevalence of physical inactivity and obesity among young adults has reached alarming levels, with university students particularly vulnerable to declining physical fitness and health (1). This trend is especially concerning as the university period represents a critical developmental stage that often establishes lifelong health habits (2).

In China, the physical fitness and health status of college students has become a matter of national concern. Since 2007, China has implemented the National Student Physical Fitness and Health Standard (NSPFHS), requiring all university students to undergo annual comprehensive physical health assessments (3). These standardized tests evaluate multiple aspects including body mass index (BMI), vital capacity, speed, endurance, strength, and flexibility, which collectively assess students’ overall physical health status rather than merely their athletic abilities. The implementation of uniform measurement and evaluation criteria has generated large-scale standardized data, providing a valuable foundation for comprehensive analysis of student physical health trends across multiple dimensions of wellness.

BMI has been widely adopted as a preliminary screening tool for weight status assessment due to its simplicity and established correlation with health outcomes (4). Recent debates, including the WHO 2022 revisions, highlight both the utility and the shortcomings of BMI in health monitoring. While BMI remains a practical screening tool, it has notable limitations, including its inability to distinguish between fat and muscle mass (5). Based on prior evidence, we hypothesized that students with “normal BMI but poor fitness” would emerge as a hidden high-risk group, while some overweight students might still demonstrate good fitness. Physical Fitness and Health (PFAH) is a comprehensive concept that goes beyond mere physical fitness, which encompasses a broader spectrum of health indicators including physiological functions, body composition, and overall health status (6). International research has consistently demonstrated that higher PFAH levels are associated with enhanced cognitive function, academic performance, and psychological well-being (7–9). A comprehensive PFAH assessment provides a more holistic view of students’ overall health status beyond isolated physical abilities.

The development of targeted interventions for improving college students’ health requires precise identification of different at-risk populations and their specific characteristics. Current generalized approaches to physical education in Chinese universities often fail to address the unique needs of diverse student populations (10).

This study proposes a novel BMI-PFAH cross-classification framework that integrates body mass index and comprehensive physical fitness and health assessment into a holistic evaluation system. By cross-classifying students based on BMI categories (normal, overweight, obese) and PFAH levels (good, pass, fail) that incorporate multiple health-related parameters beyond mere physical performance, we can identify distinct groups with potentially different health risk profiles and intervention needs.

The primary objectives of this study are to: (1) establish and validate a BMI-PFAH cross-classification framework to identify distinct risk groups among Chinese college students; (2) characterize high-risk populations and their specific fitness deficiencies; (3) validate effective predictive indicators for identifying these high-risk groups; and (4) offer practical guidance for designing and implementing specific improvement measures tailored to different student groups’ needs in higher education settings.

## Materials and methods

### Study design

This study employed a cross-sectional research design to evaluate Chinese college students’ physical fitness and health, identify group differences and high-risk populations through BMI-PFAH cross-classification, and validate predictive indicators to inform health improvement strategies.

### Participants

This study included 3,026 Chinese college students (1,435 men, 1,591 women, aged 18–22 years) from three public universities in eastern China (two in Henan, one in Zhejiang) in 2024. A stratified cluster sampling method was used, selecting one department per university and randomly sampling classes within them. All eligible students in selected classes were invited (response rate: 92.4%). The sample represents undergraduate students in eastern China’s public universities, aligning with national demographics. However, it may underrepresent students from rural or western regions, where lifestyle and physical activity patterns could differ substantially. This limits the generalizability of our findings, which should be interpreted primarily within the context of eastern China.

#### Inclusion criteria

Enrolled undergraduate students in their freshman, sophomore, or junior years; Completed the full battery of National Student Physical Fitness & Health tests.

#### Exclusion criteria

Students with incomplete test data or missing BMI measurements; Students with medical conditions or injuries that prevented participation in tests; Senior students or postgraduates, to focus on a homogenous undergraduate cohort.

### Ethical approval and consent

This study was approved by the Institutional Review Board (IRB) of Zhumadian Preschool Education College, ensuring ethical oversight of all research procedures involving human participants. All experiments were conducted in strict accordance with the Declaration of Helsinki and the guidelines set forth by the Ministry of Education of the People’s Republic of China for research involving student populations. Informed consent was obtained from all participants prior to their involvement in the study. Participants were provided with detailed information about the study’s purpose, procedures, and their right to withdraw at any time without consequences. For participants under the age of 20, additional consent was obtained from their legal guardians to ensure compliance with ethical standards.

### Data collection

**Figure 1:**
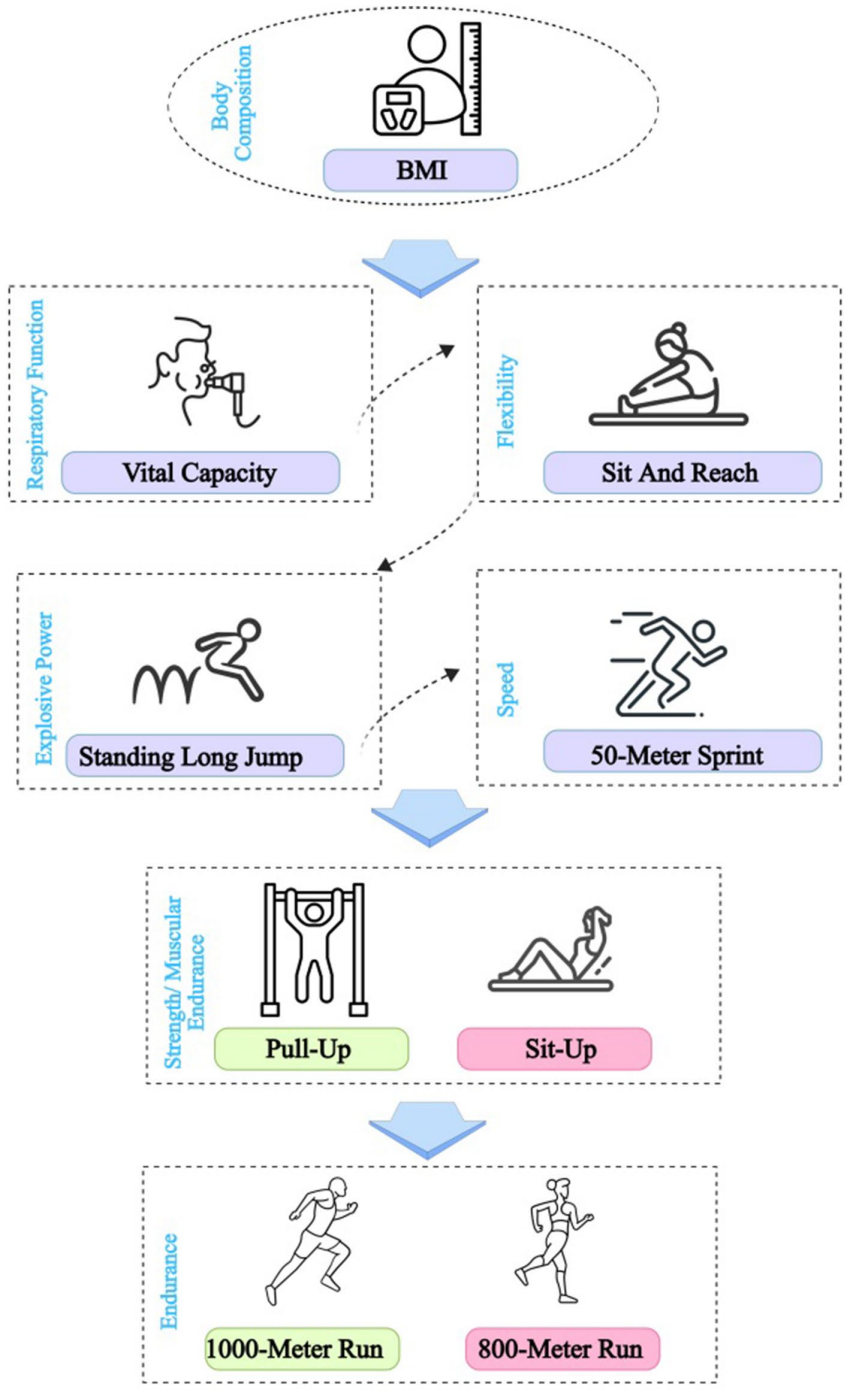
Physical fitness and health test flow chart.

In China, undergraduate students in regular higher education institutions are required to take Physical Fitness and Health (PFAH) Test every academic year (3). Standardized PFAH Test were used to assess physical fitness and health, administered per National Student Physical Fitness and Health Standard protocols by trained personnel, including the following components (Figure 1) (11):

Body Composition: BMI. BMI is calculated from height and weight: BMI= $\frac{\text{weight}\;(\mathrm{kg})}{\text{height}\;{\left(m\right)}^{2}}$ . BMI categorized as normal (<25 kg/m^2^), overweight (25–29.9 kg/m^2^), and obesity (≥30 kg/m^2^).
Respiratory function: Vital Capacity (VC). Vital capacity is measured using a spirometer, where participants maximally inhale and forcefully exhale, with the largest volume recorded in milliliters (ml) from multiple trials.
Speed: 50-meter sprint. The 50-meter sprint is measured by timing participants as they run a straight 50-meter distance at maximum speed, typically using an electronic timing system, with the fastest time recorded in seconds (s).
Explosive Power: Standing long jump (SLJ). The SLJ is measured by having participants jump forward from a stationary position with both feet, recording the farthest distance from the starting line to the landing point in centimeters (cm).
Flexibility: Sit-and-reach (SAR). The SAR is measured by having participants sit with legs extended and reach forward as far as possible along a standardized box, recording the maximum distance in centimeters (cm).
Endurance: 1000-meter run (men) or 800-meter run (women). The 1,000-meter or 800-meter run is measured by timing participants as they complete the designated distance on a standard track at maximum effort, recording the time in seconds (s).
Strength/Muscular Endurance: Pull-up (men) or sit-up (women). Pull-up or sit-up are measured by counting the maximum number of consecutive repetitions completed with proper form within one minute, recorded as the number of repetitions (*N*).
Scoring: Standard Score = BMI individual score × 15% + Vital Capacity individual score × 15% + 50-meter sprint individual score × 20% + Sit-and-reach flexibility individual score × 10% + Standing long jump individual score × 10% + 1-min Pull-ups (men)/ sit-ups (women) individual score × 10% + 1,000-meter run (men)/800-meter run (women) individual score × 20%. The Physical Fitness and Health (PFAH) score classified as Good (≥80), Pass (60–79), or Fail (<60) (12).

### Cross-classification framework

Combined BMI categories (normal, overweight, obese) with PFAH levels (Good, Pass, Fail) to form eight groups (Table 1):

**Table 1:** Nomenclature of BMI and Physical Fitness & Health cross stratification.

PFAH classification	BMI classification		
	Normal	Overweight	Obesity
PFAH-Good	Group A: Normal-Good	Group B: Overweight-Good	/
PFAH-Pass	Group C: Normal-Pass	Group D: Overweight-Pass	Group E: Obesity-Pass
PFAH-Fail	Group F: Normal-Fail	Group G: Overweight-Fail	Group H: Obesity-Fail

BMI, Body Mass Index; PFAH, Physical Fitness and Health; Obesity-Good excluded due to low prevalence (*n* = 0); Due to the small number of underweight individuals (*n* = 47), this study did not include them in the analysis.

Due to the small number of underweight individuals (*n* = 47, <2% of the total sample), this study did not include them in the analysis to avoid unstable estimates. However, we recognize that underweight students may also face distinct health risks. Future research should include larger samples of underweight students to capture the full spectrum of risk profiles.

High-Risk Group: The high-risk groups (E, F, G, H) are defined as students with combinations of obesity or normal/overweight BMI and poor PFAH performance, specifically obesity-pass (E), normal-fail (F), overweight-fail (G), and obese-fail (H).

### Statistical analysis

Participant characteristics (e.g., height, weight, and PFAH performance) summarized as means ± standard deviations for continuous variables and frequencies (%) for categorical variables. Independent samples *t*-tests (for continuous variables) and chi-square tests (for categorical variables) were used to compare BMI, PFAH test results, and composite scores between men and women. One-way ANOVA (for continuous variables) or chi-square tests (for categorical variables) were used to compare PFAH performance across academic years (freshman, sophomore, junior). The distribution of BMI and PFAH performance categories was analyzed, and chi-square tests were used to compare differences in group proportions by gender and academic year. *T*-tests were used to compare PFAH indicators between high-risk groups and low-risk groups to identify risk factors. Receiver Operating Characteristic (ROC) curve analysis was conducted to evaluate the predictive ability of standardized PFAH indicators for identifying high-risk groups, with Area Under the Curve (AUC) and 95% confidence intervals calculated. Heatmaps and radar charts were used to illustrate group differences in standardized PFAH indicators (z-scores), highlighting weaknesses in high-risk groups. The significance level was set at *p* < 0.05. Statistical analyses performed using SPSS (version 27.0) and R (version 4.3) for data processing, visualization, and modeling.

## Results

### Participant characteristics of Physical Fitness and Health

After excluding the underweight group with a small sample size (*n* = 47), this study involved 3,026 Chinese college students (1,435 men, 1,591 women) aged 18 to 22. Men had a higher BMI (22.93 ± 3.68 vs. 21.13 ± 2.91, *p* < 0.001) than women (Table 2). Most had a normal BMI (78.7%; men: 69.3%, women: 87.9%), but men had higher rates of overweight (21.4% vs. 9.1%) and obesity (9.3% vs. 3.0%, *p* < 0.001).

**Table 2:** Basic characteristics of the participants and results of the Physical Fitness & Health tests.

Indicators	Total (*n* = 3,026)	Men (*n* = 1,435)					Women (*n* = 1,591)					*P* ^b^
		Total	Freshman (*n* = 450)	Sophomore (*n* = 498)	Junior (*n* = 487)	*P* ^a^	Total	Freshman (*n* = 580)	Sophomore (*n* = 489)	Junior (*n* = 522)	*P* ^a^	
Height, cm	170.27 ± 8.35	176.75 ± 5.91	175.9 ± 5.89	176.87 ± 5.80	177.40 ± 5.95	<0.001	164.43 ± 5.41	163.29 ± 5.15	164.76 ± 5.37	165.38 ± 5.50	<0.001	0.000
Weight, kg	64.09 ± 13.04	71.74 ± 12.81	70.20 ± 11.72	71.71 ± 12.74	73.19 ± 13.68	0.002	57.19 ± 8.65	55.87 ± 7.47	57.99 ± 8.87	57.90 ± 9.47	<0.001	<0.001
BMI, kg/m^2^	21.99 ± 3.42	22.93 ± 3.68	22.66 ± 3.40	22.89 ± 3.67	23.21 ± 3.92	0.066	21.13 ± 2.91	20.94 ± 2.50	21.34 ± 2.92	21.16 ± 3.28	0.075	<0.001
Normal, *n* (%)	2,392 (78.7%)	994 (69.3%)	328 (72.89%)	340 (68.27%)	326 (66.94%)	0.120	1,398 (87.9%)	525 (90.52%)	421 (86.09%)	452 (86.59%)	0.048	<0.001
Overweight, *n* (%)	452 (14.9%)	307 (21.4%)	89 (19.78%)	110 (22.09%)	108 (22.18%)	0.601	145 (9.1%)	45 (7.76%)	54 (11.04%)	46 (8.81%)	0.020	<0.001
Obesity, *n* (%)	182 (6.0%)	134 (9.3%)	33 (7.33%)	48 (9.64%)	53 (10.88%)	0.168	48 (3.0%)	10 (1.72%)	14 (2.86%)	24 (4.60%)	0.170	<0.001
VC, ml	3889.89 ± 1023.65	4672.64 ± 816.81	4325.50 ± 691.62	5029.12 ± 834.95	4628.70 ± 754.75	<0.001	3183.53 ± 582.69	3050.91 ± 552.76	3412.82 ± 622.62	3115.72 ± 509.47	<0.001	0.000
50 m, s	8.45 ± 1.10	7.54 ± 0.69	7.58 ± 0.67	7.57 ± 0.73	7.47 ± 0.64	0.029	9.26 ± 0.68	9.34 ± 0.65	9.18 ± 0.64	9.24 ± 0.76	0.001	0.030
SLJ, cm	203.03 ± 35.03	232.79 ± 22.93	226.21 ± 18.92	235.54 ± 20.16	236.08 ± 27.34	<0.001	176.19 ± 18.45	170.74 ± 15.16	180.08 ± 17.43	178.61 ± 21.13	<0.001	<0.001
SAR, cm	17.82 ± 7.33	16.22 ± 7.92	13.80 ± 6.52	13.60 ± 6.88	21.21 ± 7.77	<0.001	19.25 ± 6.42	17.35 ± 5.72	17.67 ± 6.16	22.87 ± 5.86	<0.001	<0.001
1,000 m, s	/	257.18 ± 36.63	254.15 ± 29.74	245.49 ± 32.89	274.80 ± 41.02	<0.001	/	/	/	/	/	/
800 m, s	/	/	/	/	/	/	251.25 ± 28.86	248.13 ± 22.64	239.83 ± 26.21	267.60 ± 31.38	<0.001	/
Pull-up, *N*	/	5.81 ± 5.52	4.82 ± 4.75	5.93 ± 5.10	6.62 ± 6.40	<0.001	/	/	/	/	/	/
Sit-up, *N*	/	/	/	/	/	/	36.54 ± 8.70	33.54 ± 8.16	37.16 ± 7.44	39.31 ± 9.34	<0.001	/
PFAH-Scores	72.48 ± 10.30	69.65 ± 11.28	70.03 ± 8.64	73.02 ± 9.13	65.84 ± 13.97	<0.001	75.04 ± 8.56	75.58 ± 6.06	78.59 ± 6.11	71.10 ± 10.97	<0.001	<0.001
PFAH-Good, *n* (%)	698 (23.0%)	235 (16.4%)	61 (13.56%)	104 (20.88%)	70 (14.37%)	0.003	463 (29.1%)	141 (24.31%)	211 (43.15%)	111 (21.26%)	<0.001	<0.001
PFAH-Pass, *n* (%)	2020 (66.5%)	984 (68.6)	343 (76.22%)	357 (71.69%)	284 (58.32%)	<0.001	1,036 (65.1%)	433 (74.66%)	273 (55.83%)	330 (63.22%)	<0.001	<0.001
PFAH-Fail, *n* (%)	308 (10.1%)	216 (15.1%)	46 (10.22%)	37 (7.43%)	133 (27.31%)	<0.001	92 (5.8%)	6 (1.03%)	5 (1.02%)	81 (15.52%)	<0.001	<0.001

BMI, Body Mass Index; VC, vital capacity; SLJ, Standing long jump; SAR, Sit and Reach; PFAH, Physical Fitness and Health; *n*, number of people; *N*, number of counts; s, seconds; cm, centimeter; ml, milliliter; kg, kilogram; *P*^a^, significance of one-way ANOVA or chi-square test between grades; *P*^b^, significance of independent samples t-tests or chi-square test between genders.

Men outperformed women in vital capacity (4672.64 ± 816.81 vs. 3183.53 ± 582.69 mL), 50 m sprint (7.54 ± 0.69 vs. 9.26 ± 0.68 s), and standing long jump (232.79 ± 22.93 vs. 176.19 ± 18.45 cm), while females showed better flexibility (sit-and-reach: 19.25 ± 6.42 vs. 16.22 ± 7.92 cm, *p* < 0.001). Men averaged 257.18 ± 36.63 s for 1,000 m, women 251.25 ± 28.86 s for 800 m. PFAH scores were higher in women (75.04 ± 8.56 vs. 69.65 ± 11.28, *p* < 0.001), with 23% scoring “good” (men: 16.4%, women: 29.1%), 66.5% scoring “pass” and 10.1% scoring “fail” (men: 15.1%, women: 5.8%, *p* < 0.001).

Juniors had a higher rate of PFAH failure (men: 27.31%, women: 15.52%) and a higher rate of obesity (men: 10.88%, women:4.60%) than freshmen and sophomores (*p* < 0.001). Sophomores had the highest PFAH scores (men: 73.02 ± 9.13, women: 78.59 ± 6.11, *p* < 0.001). Most PFAH measures differed significantly between genders and grades (*p* < 0.05).

### Distribution of BMI and Physical Fitness & Health cross-classifications

Among the 3,026 students, group C (normal-pass) was the most common (52.1%), followed by A (normal-good, 22.2%) and D (overweight-pass, 11.6%). High-risk groups (E: obese-pass, 3.0%; F: normal-fail, 4.7%; G: overweight-fail, 2.5%; H: obese-fail, 3.0%) were less prevalent. Women had a higher proportion of group A (28.2% vs. 15.6%, *p* < 0.001) and group C (55.7% vs. 48.2%, *p* < 0.001) and lower rates of group D, E, G and H than men (*p* < 0.001). By grade, sophomores had the highest A group rate (30.7%), while juniors had increased F (12.1%), G (4.3%), and H (4.9%) group rates (*p* < 0.001) (Table 3).

**Table 3:** Characteristics and distributions of BMI and Physical Fitness & Health cross-classification.

Indicators	Total (*n* = 3,026)	Gender			Grade			
		Men (*n* = 1,435)	Women (*n* = 1,591)	*P*	Freshman (*n* = 1,030)	Sophomore (*n* = 987)	Junior (*n* = 1,009)	*P*
Group A, *n* (%)	673 (22.2%)	224 (15.6%)	449 (28.2%)	<0.001	198 (19.2%)	303 (30.7%)	172 (17.0%)	<0.001
Group B, *n* (%)	25 (0.8%)	11 (0.8%)	14 (0.9%)	0.731	4 (0.4%)	12 (1.2%)	9 (0.9%)	0.117
Group C, *n* (%)	1,578 (52.1%)	692 (48.2%)	886 (55.7%)	<0.001	646 (62.7%)	448 (45.4%)	484 (48.0%)	<0.001
Group D, *n* (%)	352 (11.6%)	237 (16.5%)	115 (7.2%)	<0.001	109 (10.6%)	141 (14.3%)	102 (10.1%)	0.006
Group E, *n* (%)	90 (3.0%)	55 (3.8%)	35 (2.2%)	0.008	21 (2.0%)	41 (4.2%)	28 (2.8%)	0.018
Group F, *n* (%)	141 (4.7%)	78 (5.4%)	63 (4.0%)	0.054	9 (0.9%)	10 (1.0%)	122 (12.1%)	<0.001
Group G, *n* (%)	75 (2.5%)	59 (4.1%)	16 (1.0%)	<0.001	21 (2.0%)	11 (1.1%)	43 (4.3%)	<0.001
Group H, *n* (%)	92 (3.0%)	79 (5.5%)	13 (0.8%)	<0.001	22 (2.1%)	21 (2.1%)	49 (4.9%)	<0.001

P, significance of chi-square test between genders and grades; Group A, normal BMI -good PFAH group; Group B, overweight-good PFAH group; C Group, normal BMI -pass PFAH group; Group D, overweight-pass PFAH group; Group E, obesity -pass PFAH group; Group F, normal-fail PFAH group; Group G: overweight-fail PFAH group; Group H: obesity-fail PFAH group; PFAH, Physical Fitness and Health; *n*, number of people.

### Fitness & Health test performance and weak components by cross-classification

Among 3,026 Chinese college students, Table 4 shows the Fitness & Health test characteristics by BMI and fitness cross-classification. Group A (normal-good, *n* = 673) stood out with low BMI (20.44 ± 1.71 kg/m^2^), fast 1,000 m run (226.37 ± 24.33 s) and more sit-ups (39.76 ± 10.62). B (overweight-good, *n* = 25) had stronger vital capacity (4301.56 ± 745.85 mL), faster 50 m run (8.06 ± 1.08 s), higher standing long jump (224.28 ± 40.70 cm), faster 800 m run (227.07 ± 20.68 s), longer Sit and Reach (23.06 ± 6.13) and more pull-ups (14.45 ± 3.59). High-risk groups F (normal-fail, *n* = 141), G (overweight-fail, *n* = 75), and H (obesity-fail, *n* = 92) performed poorly, with F weakest in 50 m (9.21 ± 1.27 s), G and H in strength (pull-ups: 1.66 ± 2.74/1.08 ± 3.25; sit-ups: 32.62 ± 8.16/31.07 ± 9.60), and H with the highest BMI (31.94 ± 2.96 kg/m^2^) and slowest endurance (1,000 m: 315.90 ± 57.56 s; 800 m: 317.70 ± 18.96 s).

**Table 4:** Characteristics of Physical Fitness & Health indicators by cross-classification.

Indicators	Group A (*n* = 673)	Group B (*n* = 25)	Group C (*n* = 1,578)	Group D (*n* = 352)	Group E (*n* = 90)	Group F (*n* = 141)	Group G (*n* = 75)	Group H (*n* = 92)
BMI, kg/m^2^	20.44 ± 1.71	25.41 ± 1.13	20.64 ± 1.75	25.58 ± 1.15	30.34 ± 2.49	20.79 ± 1.89	25.99 ± 1.14	31.94 ± 2.96
VC, ml	4013.85 ± 940.94	4301.56 ± 745.85	3640.83 ± 967.41	4410.00 ± 1021.99	4628.96 ± 1311.92	3753.83 ± 916.72	4092.12 ± 867.84	4472.41 ± 1036.52
50 m, s	8.21 ± 0.99	8.06 ± 1.08	8.60 ± 1.011	8.16 ± 1.06	8.28 ± 1.02	9.21 ± 1.27	8.69 ± 0.96	8.71 ± 1.04
SLJ, cm	209.52 ± 34.64	224.28 ± 40.70	198.08 ± 34.57	210.33 ± 33.09	209.54 ± 37.66	202.53 ± 40.38	200.81 ± 28.33	202.67 ± 30.00
SAR, cm	20.60 ± 6.47	23.06 ± 6.13	16.82 ± 7.06	17.06 ± 6.94	17.49 ± 6.95	19.77 ± 9.12	14.39 ± 9.46	15.96 ± 8.12
1,000 m, s	226.37 ± 24.33	238.73 ± 25.66	255.18 ± 26.37	260.67 ± 28.46	259.45 ± 25.54	310.50 ± 43.72	297.43 ± 41.82	315.90 ± 57.56
800 m, s	231.96 ± 20.47	227.07 ± 20.68	256.99 ± 26.02	260.56 ± 27.39	277.31 ± 23.23	306.06 ± 33.01	301.86 ± 21.81	317.70 ± 18.96
Pull-up, N	13.19 ± 4.50	14.45 ± 3.59	5.27 ± 0.47	3.78 ± 3.952	3.05 ± 3.89	4.16 ± 4.90	1.66 ± 2.74	1.08 ± 3.25
Sit-up, N	39.76 ± 10.62	38.27 ± 15.82	34.37 ± 8.15	35.17 ± 8.29	35.97 ± 10.80	36.85 ± 10.07	32.62 ± 8.16	31.07 ± 9.60

Group A, normal BMI -good PFAH group; Group B, overweight-good PFAH group; C Group, normal BMI -pass PFAH group; Group D, overweight-pass PFAH group; Group E, obesity -pass PFAH group; Group F, normal-fail PFAH group; Group G: overweight-fail PFAH group; Group H: obesity-fail PFAH group; PFAH, Physical Fitness and Health; BMI, Body Mass Index; VC, vital capacity; SLJ, Standing long jump; SAR, Sit and Reach; *n*, number of people; *N*, number of counts; s, seconds; cm, centimeter; ml, milliliter; kg, kilogram; n, number of people.

**Figure 2:**
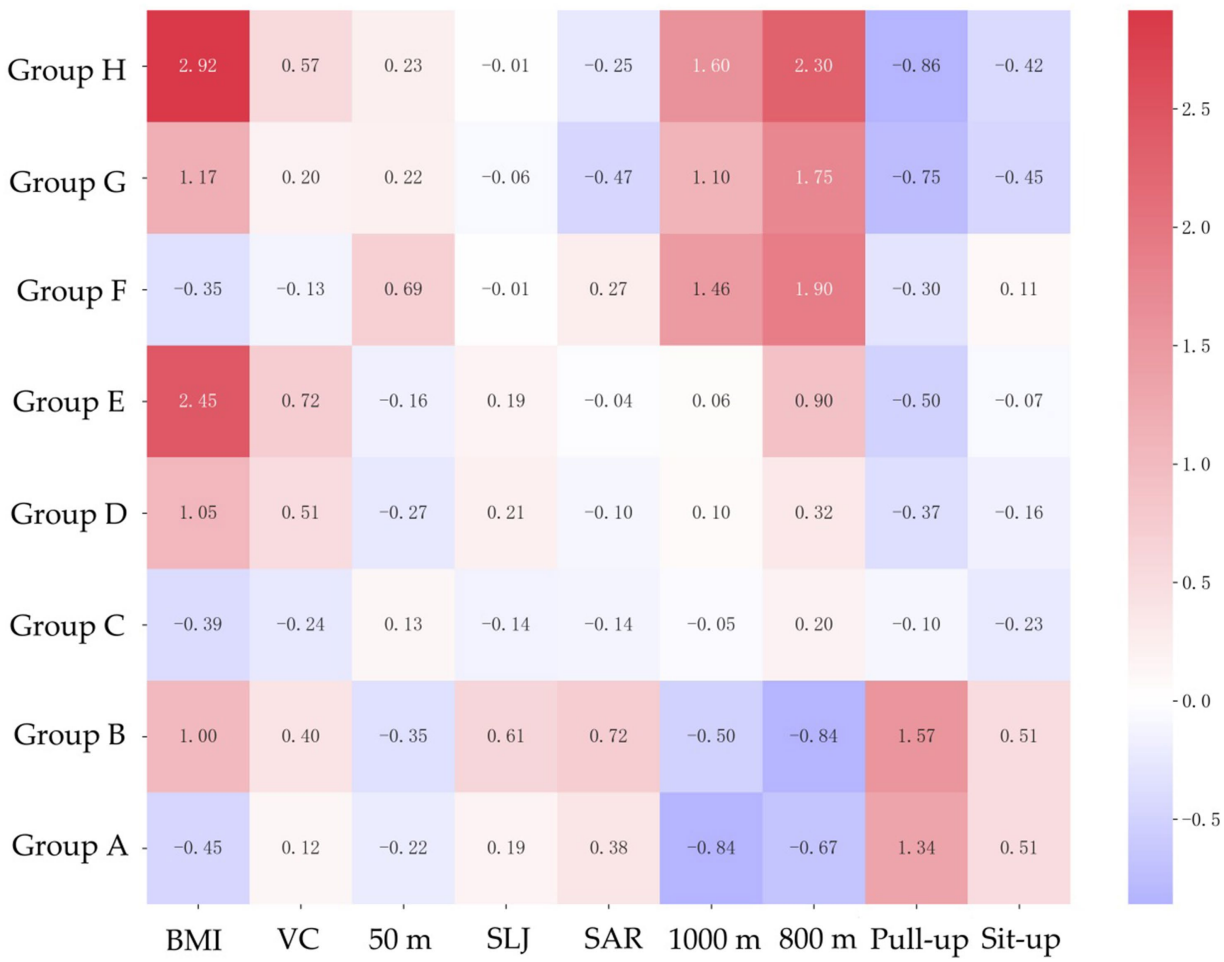
Characteristics of Physical Fitness & Health indicators based on standardized data. Group A, normal BMI -good PFAH group; Group B, overweight-good PFAH group; C Group, normal BMI -pass PFAH group; Group D, overweight-pass PFAH group; Group E, obesity -pass PFAH group; Group F, normal-fail PFAH group; Group G: overweight-fail PFAH group; Group H: obesity-fail PFAH group; PFAH, Physical Fitness and Health; BMI, Body Mass Index; VC, vital capacity; SLJ, standing long jump; SAR, Sit and Reach.

The heat map (Figure 2) of standardized scores highlights weaknesses. Groups A and B perform above average in most tests. High-risk groups E, F, G and H had significant weaknesses: E in BMI (z = 2.45), F in speed (50 m: z = 0.69), G in flexibility (Sit and Reach: z = −0.47) and muscle endurance (sit-up: z = −0.45), and H in cardiopulmonary endurance (1,000 m: z = 1.60; 800 m: z = 2.30) and muscle strength (pull-up: z = −0.86), confirming their fitness deficits across multiple domains.

### Gender-specific Fitness & Health test performance and weak components by cross-classification

Table 5 shows Physical Fitness & Health test results for 3,026 students across cross-classified groups, stratified by gender. The results by gender were similar to the previous ones, but there were still differences. The speed (50 m) of group A was the fastest of the groups (men: 7.01 ± 0.48 s, women: 8.80 ± 0.54 s); the vital capacity of men in group F (4319.85 ± 739.97 mL) and women in group G (2939.40 ± 420.40 mL) was the worst; the 50 m performance of women in group F (10.44 ± 0.59 s) and men in group H (8.48 ± 0.92 s) was the worst; and the standing long jump of the girls in group G and the boys in group H was the worst.

**Table 5:** Characteristics of Physical Fitness & Health indicators by cross-classification for different genders.

Indicators	A Group (*n* = 673)		B Group (*n* = 25)		C Group (*n* = 1,578)		D Group (*n* = 352)		E Group (*n* = 90)		F Group (*n* = 141)		G Group (*n* = 75)		H Group (*n* = 92)	
	Men	Women	Men	Women	Men	Women	Men	Women	Men	Women	Men	Women	Men	Women	Men	Women
BMI, kg/m^2^	20.72±1.63	20.31±1.73**	25.18±1.19	25.59±1.09	21.01±1.72	20.36±1.72***	25.59±1.15	25.56±1.15	30.06±2.52	30.79±2.40	21.45±1.67	19.97±1.84***	26.06±1.10	25.74±1.27	31.82±2.93	32.69±3.18
VC, ml	5007.9±758.65	3517.89 ± 548.53***	5033.18±394.12	3726.71 ± 314.19***	4480.55±758.63	2985.93 ± 487.80***	4919.34±735.16	3360.32 ± 667.97***	5393.15±1017.70	3428.09 ± 656.65***	4319.85±739.97	3030.08 ± 530.23***	4390.24±683.98	2939.40 ± 420.40***	4665.84±924.45	3297.00 ± 921.59***
50 m, s	7.01±0.48	8.80±0.54***	7.02±0.43	8.89±0.59***	7.52±0.53	9.44±0.62**	7.58±0.64	9.36±0.70***	7.57±0.49	9.396±0.54***	8.47±0.94	10.44±0.59***	8.39±0.64	10.18±0.97***	8.48±0.92	10.22±0.30***
SLJ, cm	251.08 ± 20.20	188.78 ± 17.39***	260.91±15.09	195.50 ± 29.25***	232.52±17.94	171.19 ± 15.07**	230.02±18.73	169.75 ± 13.37***	234.53 ± 19.19	170.29 ± 23.08***	226.75±33.59	172.44 ± 24.93***	210.12±22.88	164.20 ± 14.65***	208.99±26.73	164.77 ± 18.73***
SAR, cm	19.76±7.88	21.03±5.59*	23.97±5.75	22.35±6.54	14.94±7.36	18.29±6.46***	16.43±7.36	18.36±5.81*	16.79±7.37	18.59 ± 6.17	17.51±10.35	22.46±6.51***	14.68 ± 9.08	13.38±10.97	15.15±7.86	21.26±8.11*
1,000 m, s	226.37 ± 24.33	/	238.73 ± 25.66	/	255.18 ± 26.37	/	260.67 ± 28.46	/	259.45 ± 25.54	/	310.50 ± 43.72	/	297.43 ± 41.82	/	315.90 ± 57.56	/
800 m, s	/	231.96 ± 20.47	/	227.07 ± 20.68	/	256.99 ± 26.02	/	260.56 ± 27.39	/	277.31 ± 23.23	/	306.06 ± 33.01	/	301.86 ± 21.81	/	317.70 ± 18.96
Pull-up, N	13.19 ± 4.50	/	14.45 ± 3.59	/	5.27 ± 0.47	/	3.78 ± 3.952	/	3.05 ± 3.89	/	4.16 ± 4.90	/	1.66 ± 2.74	/	1.08 ± 3.25	/
Sit-up, N	/	39.76 ± 10.62	/	38.27 ± 15.82	/	34.37 ± 8.15	/	35.17 ± 8.29	/	35.97 ± 10.80	/	36.85 ± 10.07	/	32.62 ± 8.16	/	31.07 ± 9.60

Group A, normal BMI -good PFAH group; Group B, overweight-good PFAH group; C Group, normal BMI -pass PFAH group; Group D, overweight-pass PFAH group; Group E, obesity -pass PFAH group; Group F, normal-fail PFAH group; Group G: overweight-fail PFAH group; Group H: obesity-fail PFAH group; PFAH, Physical Fitness and Health; BMI, Body Mass Index; VC, vital capacity; SLJ, Standing long jump; SAR, Sit and Reach; *n*, number of people; *N*, number of counts; s, seconds; cm, centimeter; ml, milliliter; kg, kilogram; n, number of people; ***, significance of independent samples *t*-test between genders and *p* < 0.001; **, *p* < 0.01; *, *p* < 0.0.

**Figure 3:**
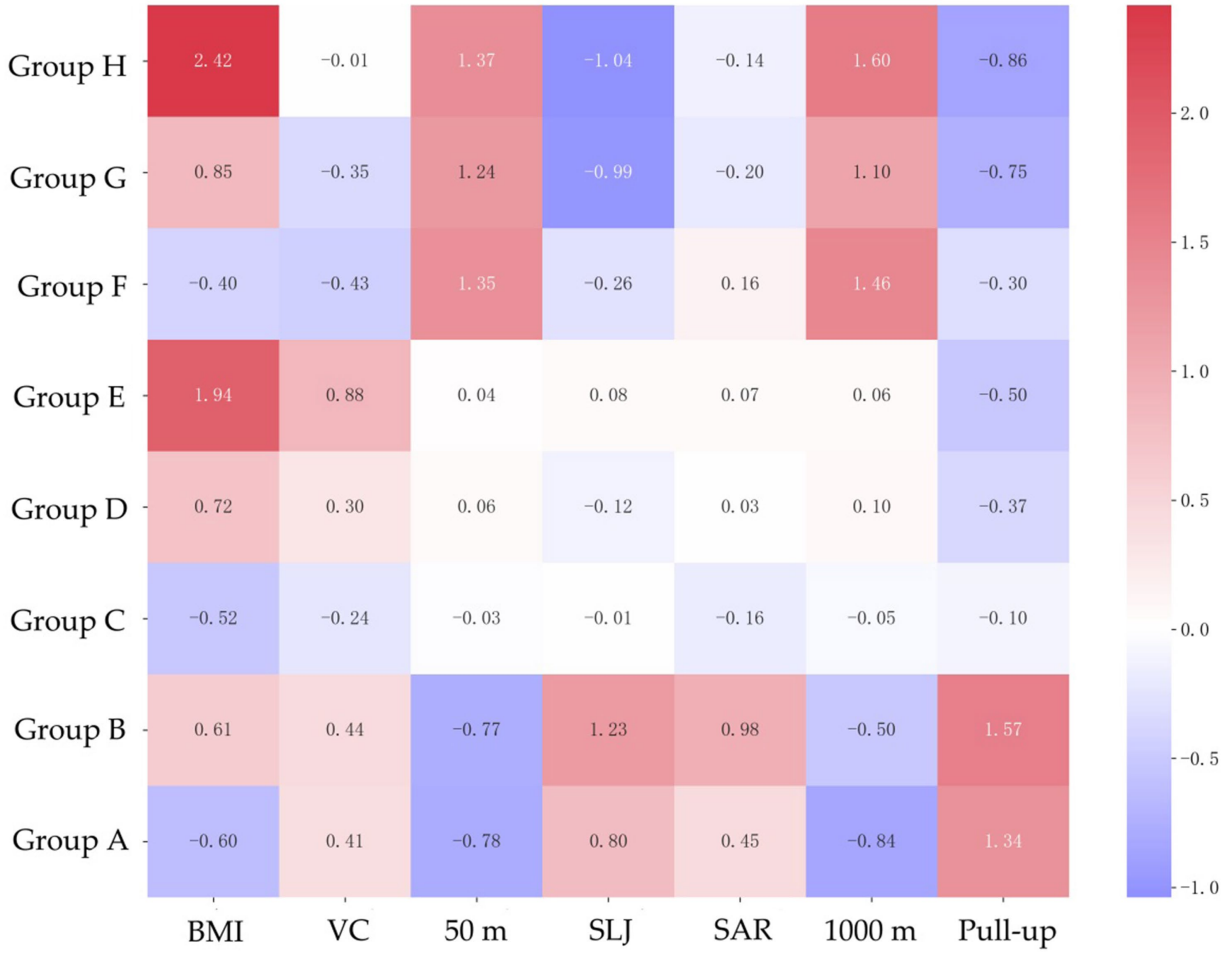
Characteristics of Physical Fitness & Health indicators based on standardized data in men.

**Figure 4:**
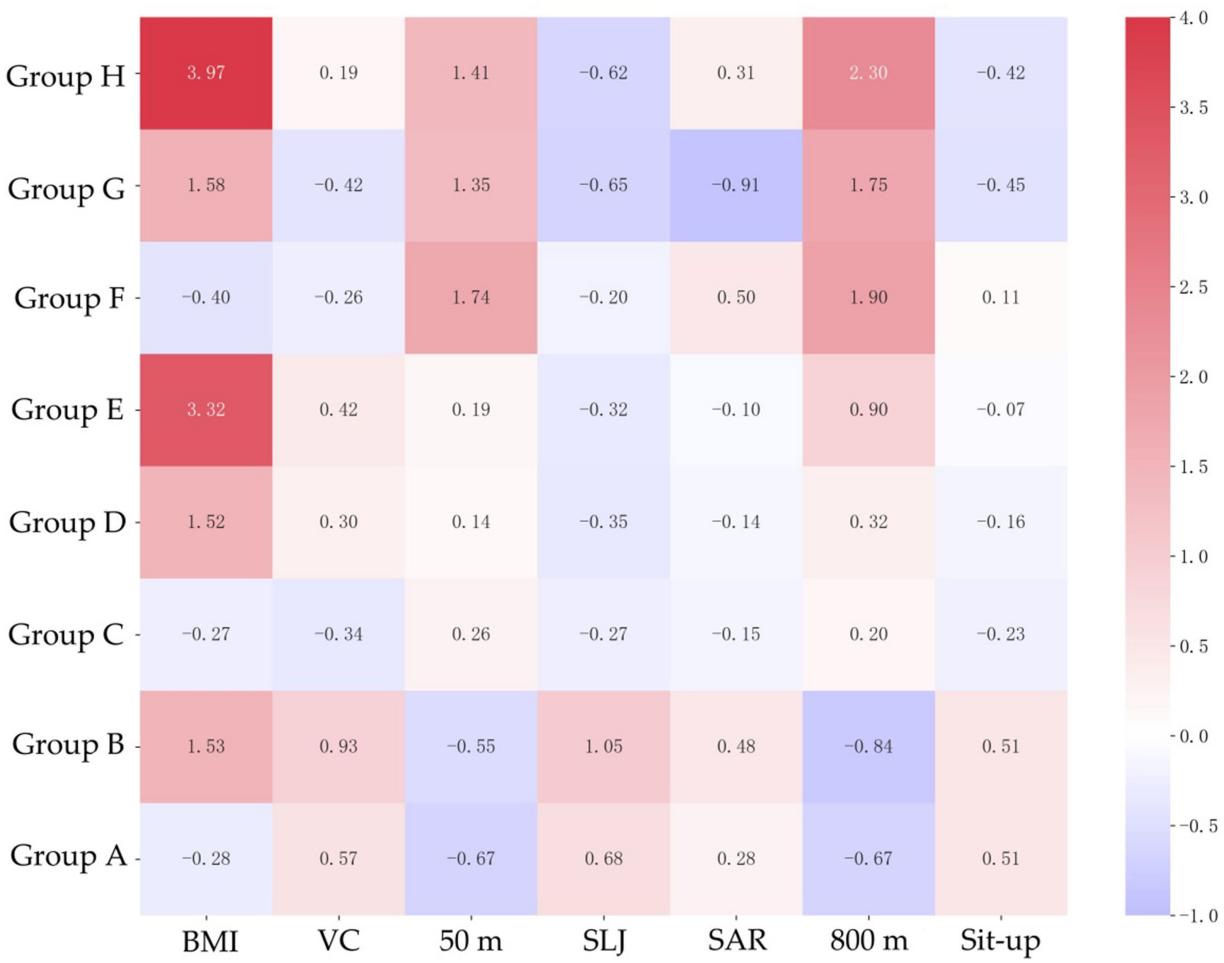
Characteristics of Physical Fitness & Health indicators based on standardized data in women.

Heatmaps (Figures 3, 4) of standardized scores highlight gender-specific weaknesses in Physical Fitness & Health items. Gender stratification made it easier to identify the characteristics of each group. For men, A and B groups scored above average, while high-risk groups E, F, G and H had significant weaknesses: E in BMI (z = 1.94), F in vital capacity (z = −0.43), G in flexibility (Sit and Reach: z = −0.20), and H in speed (50 m: z = 1.37), explosive force (standing long jump: z = −1.04), cardiopulmonary endurance (1,000 m: z = 1.60) and muscle strength (pull-up: z = −0.86). For women, the trend is similar to that of men.

### Risk factors and fitness profiles of high-risk groups

**Figure 5:**
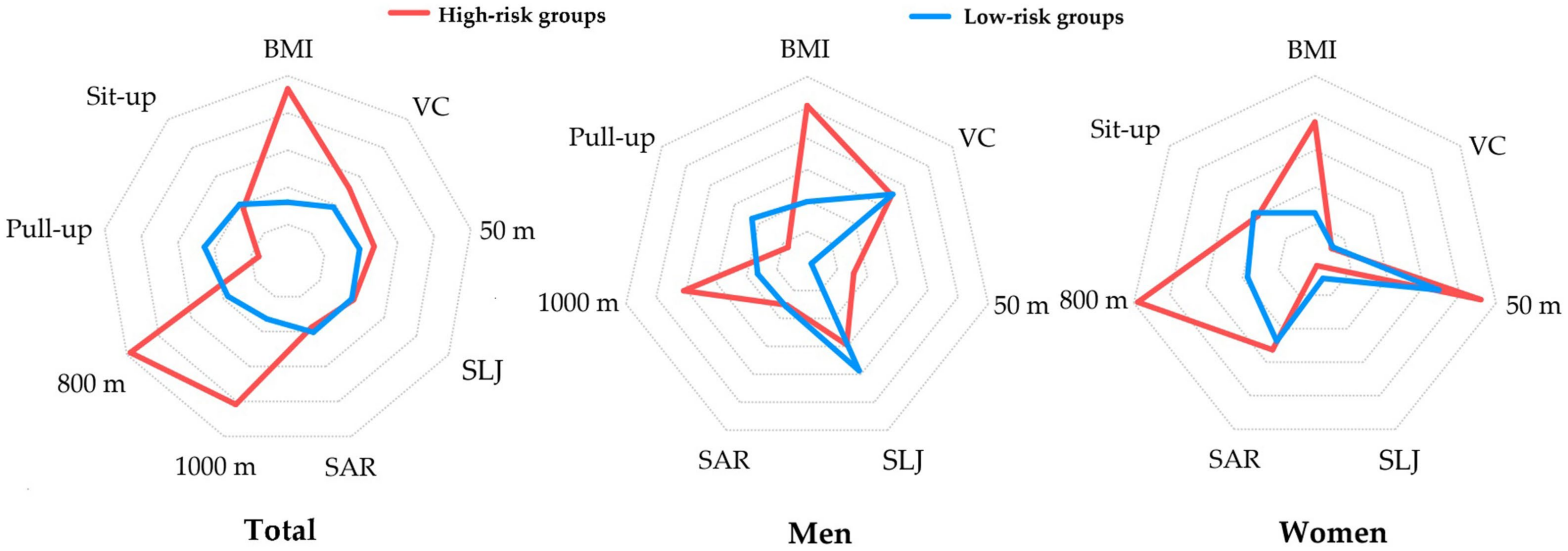
Characteristics of Physical Fitness & Health indicators in high-risk groups based on standardized data.

Table 6 compares the E, F, G, and H groups (high-risk, *n* = 271) with the low-risk group (*n* = 3,026). High-risk students had higher BMI (26.51 ± 5.17 vs. 21.30 ± 2.41 kg/m^2^), and slower 50 m sprint (8.65 ± 1.11 vs. 8.43 ± 1.01 s) and endurance runs (1,000 m: 295.40 ± 50.09 vs. 250.57 ± 29.07 s; 800 m: 292.93 ± 29.80 vs. 249.24 ± 27.26 s, *p* < 0.001). Men in high-risk groups had weaker standing long jump (219.54 ± 28.74 vs. 235.85 ± 20.17 cm) and pull-ups (2.48 ± 4.01 vs. 6.58 ± 5.54, *p* < 0.001), while women showed deficits in standing long jump (170.05 ± 22.80 vs. 176.71 ± 17.94 cm, *p* = 0.002). Vital capacity and flexibility showed no significant differences after gender stratification (*p* > 0.05). Radar charts (Figure 5) of standardized scores highlight weaknesses and confirm their fitness and health deficits in comparison to low-risk peers.

**Table 6:** Identification of risk factors in high-risk groups.

Indicators	Total (*n* = 3,026)			Men (*n* = 1,435)			Women (*n* = 1,591)		
	High-risk groups (*n* = 398)	Low-risk groups (*n* = 2,628)	*P*	High-risk groups (*n* = 271)	Low-risk groups (*n* = 1,164)	*P*	High-risk groups (*n* = 127)	Low-risk groups (*n* = 1,464)	*P*
BMI, kg/m^2^	26.51 ± 5.17	21.30 ± 2.41	<0.001	27.22 ± 4.75	21.93 ± 2.48	<0.001	24.98 ± 5.70	20.80 ± 2.24	<0.001
VC, ml	4184.20 ± 1097.02	3845.75 ± 1005.01	<0.001	4654.84 ± 933.12	4676.78 ± 787.75	0.721	3159.44 ± 629.32	3185.57 ± 578.75	0.632
50 m, s	8.65 ± 1.11	8.43 ± 1.01	0.002	8.20 ± 0.85	7.43 ± 0.58	<0.001	9.87 ± 0.75	9.23 ± 0.67	<0.001
SLJ, cm	203.84 ± 35.48	202.90 ± 34.97	0.621	219.54 ± 28.74	235.85 ± 20.17	<0.001	170.05 ± 22.80	176.71 ± 17.94	0.002
SAR, cm	17.36 ± 8.71	17.88 ± 7.10	0.263	16.05 ± 8.83	16.26 ± 7.70	0.729	20.12 ± 7.81	19.17 ± 6.28	0.188
1,000 m, s	295.40 ± 50.09	250.57 ± 29.07	<0.001	295.40 ± 50.09	250.57 ± 29.07	<0.001	/	/	/
800 m, s	292.93 ± 29.80	249.24 ± 27.26	<0.001	/	/	/	292.93 ± 29.80	249.24 ± 27.26	<0.001
Pull-up, N	2.48 ± 4.01	6.58 ± 5.5	<0.001	2.48 ± 4.01	6.58 ± 5.54	<0.001	/	/	/
Sit-up, N	35.44 ± 10.11	36.15 ± 9.42	0.417	/	/	/	35.94 ± 9.35	36.59 ± 8.64	0.424

BMI, Body Mass Index; VC, vital capacity; SLJ, Standing long jump; SAR, Sit and Reach; PFAH, Physical Fitness and Health; *n*, number of people; *N*, number of counts; s, seconds; cm, centimeter; ml, milliliter; kg, kilogram; P, significance of independent samples *t*-tests between high-risk groups and low-risk groups.

### Predictive performance of Fitness & Health indicators for high-risk men

**Figure 6:**
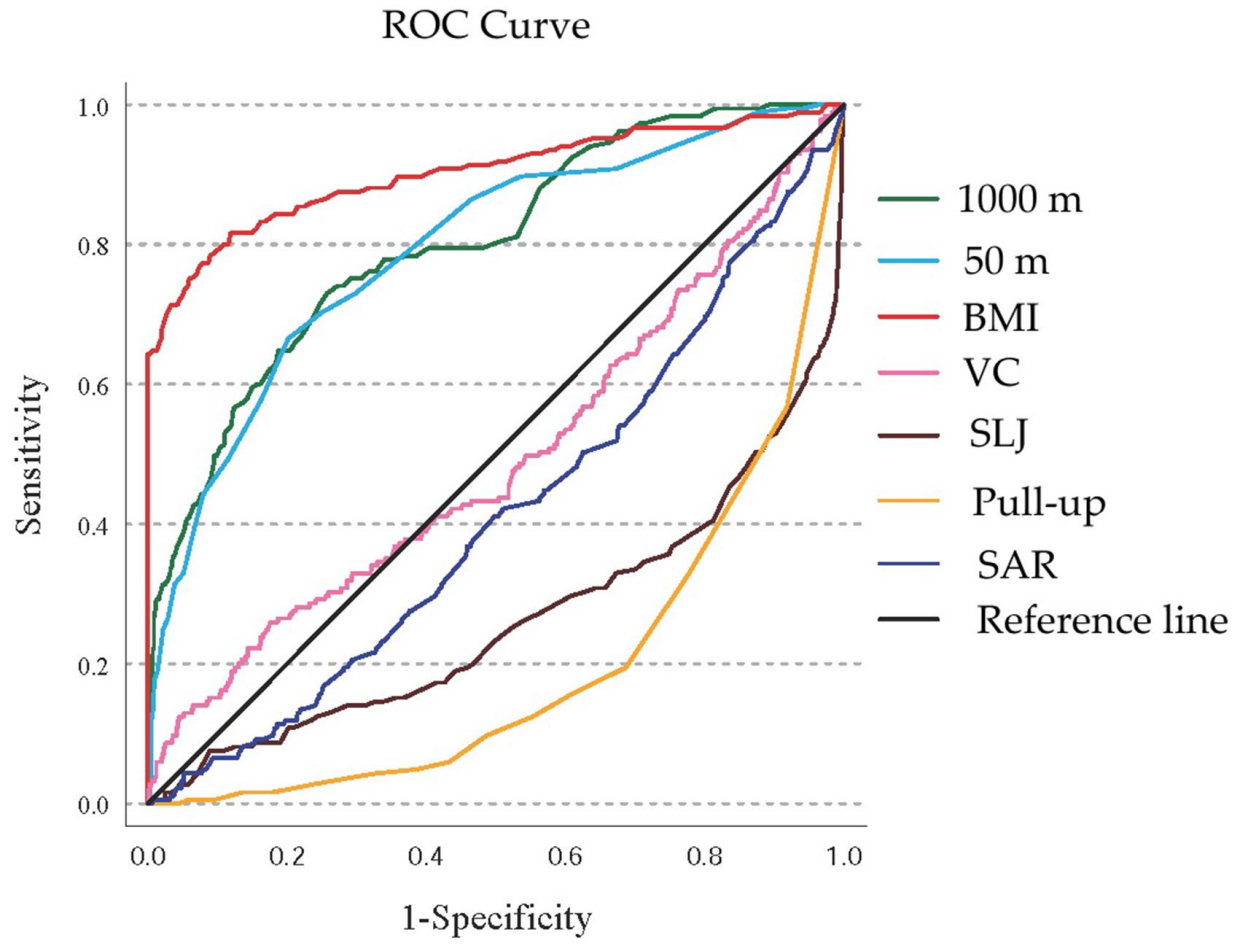
The ROC curve for predicting high-risk men. BMI, Body Mass Index; VC, vital capacity; SLJ, standing long jump; SAR, Sit and Reach; ROC, receiver operating characteristic.

ROC analysis (Figure 6) and the corresponding table (Table 7) evaluate the predictive ability of standardized Fitness & Health indicators for identifying high-risk men (groups E, F, G, and H). BMI and endurance (1,000 m) were the strongest predictors, with AUCs of 0.902 (95% CI: 0.871–0.933) and 0.801 (95% CI: 0.765–0.836), respectively (*p* < 0.001). Speed (50 m) also showed good predictive power (AUC: 0.792, 95% CI: 0.755–0.828, *p* = 0.019). The ROC curves confirm BMI, 1000 m and 50 m as the most effective indicators for identifying high-risk men. No significance was found for vital capacity (AUC: 0.494, 95% CI: 0.446–0.543, *p* = 0.820). The AUCs for the rest of the indicators were less than 0.5.

**Table 7:** AUC prediction results of each indicator for high-risk men.

Indicators	AUC	SE	*P*	95% CI	
BMI	0.902	0.016	0.000	0.871	0.933
1,000 m	0.801	0.018	0.000	0.765	0.836
50 m	0.792	0.019	0.000	0.755	0.828
SAR	0.414	0.023	0.000	0.370	0.458
SLJ	0.263	0.023	0.000	0.218	0.308
Pull-up	0.195	0.016	0.000	0.163	0.227
VC	0.494	0.025	0.820	0.446	0.543

The test outcome variables were standardized; BMI, Body Mass Index; VC, vital capacity; SLJ, Standing long jump; SAR, Sit and Reach; AUC, area under the ROC curve; ROC, receiver operating characteristic; SE, standard error; CI, Confidence Interval.

### Predictive performance of Fitness & Health indicators for high-risk women

**Figure 7:**
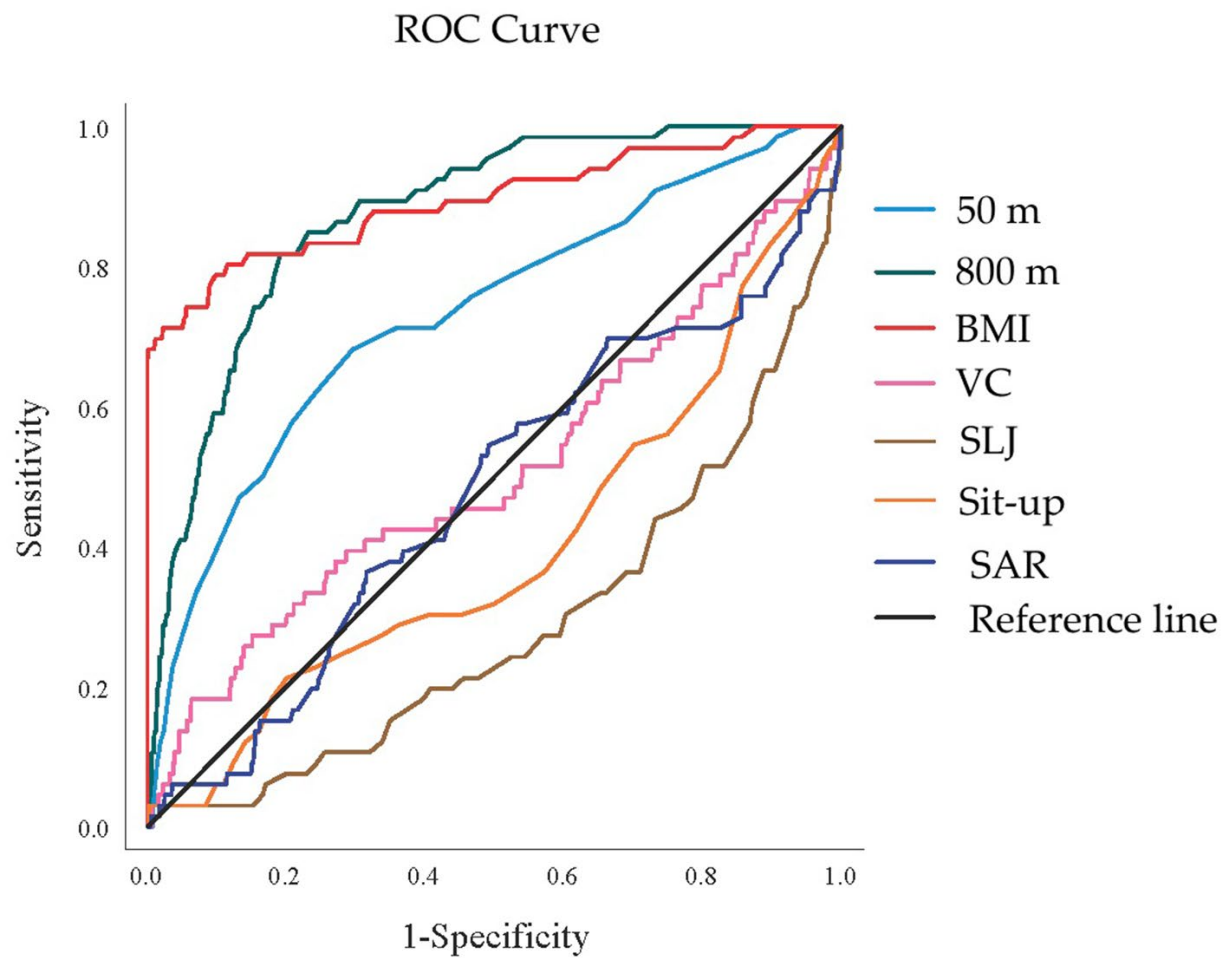
The ROC curve for predicting high-risk women. BMI, Body Mass Index; VC, vital capacity; SLJ, standing long jump; SAR, Sit and Reach; ROC, receiver operating characteristic.

ROC analysis (Figure 7) and the corresponding table (Table 8) assess the predictive ability of standardized Fitness & Health indicators for identifying high-risk women (groups E, F, G, and H). BMI and endurance (800 m) were the strongest predictors, with AUCs of 0.895 (95% CI: 0.842–0.947) and 0.874 (95% CI: 0.836–0.912), respectively (*p* < 0.001). Speed (50 m) also showed good predictive power (AUC: 0.731, 95% CI: 0.664–0.798, *p* < 0.001). The ROC curves confirm BMI, 800 m and 50 m as the most effective indicators for identifying high-risk women. No significance was found for vital capacity (AUC: 0.513, 95% CI: 0.431–0.594, *p* = 0.757) and flexibility (Sit and Reach, AUC: 0.478, 95% CI: 0.402–0.554, *p* = 0.565). The AUCs for the rest of the indicators were less than 0.5.

**Table 8:** AUC prediction results of each indicator for high-risk women.

Indicators	AUC	SE	*P*	95% CI	
BMI	0.895	0.027	0.000	0.842	0.947
800 m	0.874	0.019	0.000	0.836	0.912
50 m	0.731	0.034	0.000	0.664	0.798
SLJ	0.289	0.034	0.000	0.221	0.356
Sit-up	0.402	0.038	0.011	0.327	0.478
VC	0.513	0.042	0.757	0.431	0.594
SAR	0.478	0.039	0.565	0.402	0.554

The test outcome variables were standardized; BMI, Body Mass Index; VC, vital capacity; SLJ, Standing long jump; SAR, Sit and Reach; AUC, area under the ROC curve; ROC, receiver operating characteristic; SE, standard error; CI, Confidence Interval.

## Discussion

This study established a novel cross-classification framework integrating BMI and Physical Fitness and Health (PFAH) assessment to identify distinct risk groups among Chinese college students. Our findings provide a comprehensive understanding of physical health status beyond traditional single-metric approaches, revealing critical insights about diverse risk profiles and specific fitness deficiencies among university students.

### Key findings and significance of the cross-classification framework

Our study demonstrates that the BMI-PFAH cross-classification framework effectively identifies distinct risk groups with unique characteristics that would be overlooked by traditional single-metric assessments. While the majority of students (52.1%) fell into the normal-pass category (Group C), we identified several high-risk groups with concerning fitness profiles: obesity-pass (Group E, 3.0%), normal-fail (Group F, 4.7%), overweight-fail (Group G, 2.5%), and obesity-fail (Group H, 3.0%). These findings highlight the critical importance of comprehensive assessment approaches that integrate both body composition and PFAH parameters.

The traditional reliance on BMI alone as a health indicator has significant limitations, as demonstrated by the substantial proportion of students with normal BMI but failed PFAH scores (Group F). This “hidden risk” population would be categorized as healthy in weight-focused screening programs despite exhibiting poor physical fitness, potentially delaying needed interventions. Conversely, some overweight students demonstrated good physical fitness (Group B), suggesting that excess weight does not invariably predict poor fitness, particularly in individuals with higher muscle mass.

### Characteristics and mechanisms underlying high-risk groups

#### Group E (obesity-pass)

Students in the obesity-pass group maintained adequate fitness levels despite elevated BMI. This finding aligns with the “metabolically healthy obesity” phenomenon documented in previous research (13). These individuals likely engage in regular physical activity that preserves cardiorespiratory fitness and muscular strength despite excess adiposity. The long-term health trajectory of this group merits further investigation, as some studies suggest that metabolically healthy obesity may represent a transient state rather than a stable condition (14).

#### Group F (normal-fail)

Perhaps most concerning is the identification of students with normal BMI but failed PFAH scores. This group exemplifies the “normal weight obesity” phenomenon characterized by inadequate muscle mass and excessive body fat despite normal BMI (15). Their most pronounced deficiency was in speed (50 m sprint), suggesting particular impairments in anaerobic capacity, neuromuscular efficiency, and explosive power. These patterns may be consistent with the “normal weight obesity” phenomenon (16), but without body composition or dietary data, such explanations remain speculative. Future studies incorporating bioimpedance or dual-energy X-ray absorptiometry (DXA) alongside nutrition assessments are needed to verify these mechanisms.

#### Groups G and H (overweight/obesity-fail)

Students in the overweight-fail and obesity-fail groups demonstrated comprehensive fitness deficiencies, with particularly poor performance in strength, flexibility (Group G), and endurance (Group H). Group H had the slowest endurance times (1,000 m: 315.90 ± 57.56 s; 800 m: 317.70 ± 18.96 s) and weakest upper body strength (pull-ups: 1.08 ± 3.25), indicating severe cardiovascular and muscular impairments. These findings align with established literature on the detrimental effects of excess adiposity on physical performance and emphasize the multifaceted impact of obesity and physical fitness on various fitness domains (17, 18).

### Gender-specific considerations

Our results revealed significant gender differences in both BMI-PFAH distribution and specific fitness deficiencies. Women demonstrated a higher prevalence of normal-good classifications (28.2% vs. 15.6% in men) and lower rates in high-risk categories, particularly in Groups G and H. These gender disparities may reflect biological differences in body composition, muscle mass distribution, and hormonal influences on physical performance (19).

The gender-stratified analysis highlighted distinct patterns of fitness impairments. Among high-risk men, deficiencies were particularly pronounced in upper body strength (pull-ups) and explosive power (standing long jump), while high-risk women showed more significant impairments in cardiorespiratory endurance. These differences likely reflect both physiological factors and gender-specific physical activity patterns, with men traditionally engaging more in strength-based activities and women in endurance or flexibility-focused exercises (20–22).

These gender-specific patterns have important implications for intervention design, suggesting that programs for high-risk male students should particularly emphasize upper body strength development and explosive power training, while interventions for females may need greater focus on improving cardiorespiratory endurance.

### Academic year patterns and educational implications

The significant differences in BMI-PFAH distributions across academic years reveal concerning trends in student health trajectories during university education. Most notably, the proportion of students in high-risk groups (particularly F, G, and H) increased substantially by junior year, with Group F (normal-fail) rising dramatically to 12.1% from approximately 1% in lower grades. However, as this is a cross-sectional study, these differences may reflect cohort effects rather than true longitudinal deterioration. Therefore, our findings should be interpreted as associations only, and future longitudinal follow-up studies are needed to confirm whether physical fitness indeed declines as students progress through university. While some of the observed differences reached statistical significance, effect sizes were small in certain comparisons. These results should therefore be interpreted with caution regarding their practical significance.

This deterioration in physical fitness as students’ progress through university aligns with previous research demonstrating declining physical activity levels during higher education (23, 24). Several factors may contribute to this trend, including increased academic pressures, reduced participation in organized sports, transition to more sedentary study patterns, and lifestyle changes related to independence and stress management (25).

These findings have profound implications for university physical education policies. Current approaches typically feature more intensive physical education requirements in early university years, with diminishing structured physical activity opportunities for upper-class students. Our results suggest this approach may be insufficient, as the need for physical activity support and intervention appears to increase rather than decrease as students’ progress through their academic careers. Universities should consider implementing continuous physical education requirements throughout all academic years, with particular attention to maintaining fitness levels among junior and senior students.

### Predictive value of fitness indicators for risk classification

The ROC analysis identified BMI, endurance capacity (1,000 m/800 m run), and speed (50 m sprint) as the most effective predictors for identifying high-risk students. For men, BMI (AUC: 0.902) and 1,000 m run (AUC: 0.801) demonstrated excellent discriminatory power, while similar patterns were observed for women with BMI (AUC: 0.895) and 800 m run (AUC: 0.874).

These findings suggest that a simplified screening approach utilizing these three parameters could efficiently identify students requiring more comprehensive assessment and intervention. This streamlined approach could significantly enhance the feasibility of large-scale screening programs while maintaining adequate sensitivity for detecting high-risk individuals. Thus, while BMI showed the strongest predictive power in ROC analysis (26), the added value of our framework lies in combining BMI with physical fitness indicators to provide a more nuanced assessment that avoids overlooking hidden risks.

Interestingly, vital capacity showed no significant predictive value for high-risk classification in either gender. This finding contrasts with some previous research highlighting associations between pulmonary function and overall fitness (27). This discrepancy may reflect the complex relationship between respiratory capacity and physical performance, where adequate vital capacity may be necessary but not sufficient for overall fitness (28).

### Tailored intervention strategies for different risk groups

Our comprehensive characterization of distinct risk groups provides a foundation for developing targeted intervention strategies that address the specific deficiencies of each population:

#### For Group E (obesity-pass)

Interventions should focus on weight management while preserving existing fitness levels. This group would benefit from nutritional counseling emphasizing caloric balance and dietary quality without drastic restrictions that might compromise physical performance (29). Physical activity recommendations should include maintaining current fitness activities while gradually increasing caloric expenditure through additional low-intensity activities (30).

#### For Group F (normal-fail)

This hidden-risk group requires interventions that address fitness deficiencies despite normal weight status. Programs should emphasize progressive resistance training to build lean muscle mass and improve metabolic health, combined with high-intensity interval training to enhance anaerobic capacity and speed performance (31–33). Nutritional guidance should focus on adequate protein intake and overall dietary quality rather than caloric restriction.

#### For Groups G and H (overweight/obesity-fail)

These high-risk groups require comprehensive interventions addressing both weight management and multiple fitness deficiencies. Initial programming should emphasize low-impact aerobics activities to improve cardiorespiratory fitness while minimizing joint stress, gradually progressive resistance training to enhance muscular strength and metabolic rate (34–36). Behavioral interventions addressing motivation, self-efficacy, and overcoming psychological barriers to physical activity are particularly important for these groups.

In terms of practical implementation, universities could adapt these recommendations to institutional contexts. For example, continuous physical education requirements could be extended to junior and senior years to counteract declining fitness. Universities might also develop tailored training programs—such as anaerobic capacity enhancement modules for normal-fail students, weight management workshops for obesity-pass students, and comprehensive fitness programs for overweight/obesity-fail students. Integration of health counseling, accessible sports facilities, and student wellness programs would further enhance feasibility and effectiveness.

Gender-specific adaptations should further refine these intervention approaches, with particular emphasis on upper body strength development for men and cardiopulmonary endurance enhancement for women in high-risk categories.

Several limitations should be considered when interpreting our findings. First, the cross-sectional design limits our ability to infer causality. Longitudinal follow-up studies are needed to confirm the trajectories suggested by academic year comparisons. Second, while our sample size was substantial, the sampling methodology and specific institutional context may limit generalizability to all Chinese university students. Regional variations in physical activity patterns, dietary habits, and educational approaches may influence the distribution of risk groups in different university populations. Moreover, this study did not include important health-related variables such as dietary patterns, sleep duration, mental health, psychological stress, and physical activity beyond mandatory PE classes. These factors may partly explain differences across groups and should be incorporated into future research. Another limitation is that the assessment was based on the Chinese National Student Physical Fitness and Health Standard (NSPFHS), which, although appropriate for the domestic context, reduces comparability with international studies. Future research may benefit from integrating internationally recognized tools. On the other hand, our exclusion of underweight students (BMI < 18.5) limits our understanding of the complete spectrum of body composition and fitness relationships. Future research should expand the framework to include this population, which may have unique fitness challenges and health risks. Finally, due to sample size and distribution limitations, we were unable to test interaction effects (e.g., gender × BMI × PFAH). Future research with larger samples should explore such interactions to uncover more nuanced relationships.

## Conclusion

This study establishes the value of a novel BMI-PFAH cross-classification framework that provides a more nuanced understanding of physical health status among Chinese college students than traditional single-metric approaches. By identifying distinct risk groups with specific deficiencies, our findings provide a foundation for developing targeted interventions that address the unique needs of diverse student populations.

By advancing beyond simplistic BMI-based categorizations to a multidimensional understanding of physical health, this framework offers valuable guidance for enhancing university physical education policies, developing targeted intervention strategies, and ultimately improving the long-term health trajectories of college students.

## Data Availability

The raw data supporting the conclusions of this article will be made available by the authors, without undue reservation.
